# Unusual Presentation of Recurrent Gallstone Ileus: A Case Report and Literature Review

**DOI:** 10.1155/2019/8907068

**Published:** 2019-10-03

**Authors:** Osayande Osagiede, Paula Pacurari, Dorin Colibaseanu, Nezar Jrebi

**Affiliations:** ^1^Mayo Clinic, Department of Surgery, Jacksonville, FL, USA; ^2^West Virginia University School of Medicine, Morgantown, WV, USA; ^3^West Virginia University, Department of Surgery, Morgantown, WV, USA

## Abstract

**Background:**

Gallstone ileus (GSI) is a rare form of small bowel obstruction (SBO) in patients with cholelithiasis, which is often poorly managed. Enhanced abdominal computed tomography (CT) with contrast is considered the most helpful diagnostic tool, as it is highly sensitive, specific, and accurate. We report an interesting case of recurrent GSI that was not detected by CT but diagnosed intraoperatively.

**Case Presentation:**

A 49-year-old female with a previous history of choledocholithiasis and ERCP presented to the emergency department following episodes of sudden cramping, epigastric pain, and nausea. An abdominal CT revealed evidence of SBO with clear evidence of GSI and a cholecystoduodenal fistula. Laparoscopic exploration of the small bowel revealed a large, calcified 3.5 cm × 3 cm gallstone with evidence of pressure necrosis; segmental bowel resection with stapled anastomosis was performed and patient recovered appropriately after surgery. Cholecystectomy was not performed due to multiple co-morbidities and absence of gallbladder stones. However, she presented two months later with signs and symptoms of SBO. A repeat abdominal CT showed dilated bowel with no clear transition point. This was suspected to be due to adhesions. After an initial conservative treatment which produced mild improvement, laparotomy was performed which revealed a second large non-calcified gallstone and necrotic small bowel with a pocket of abscess.

**Conclusion:**

The most sensitive diagnostic tool for GSI is enhanced abdominal CT but dilemma arises when GSI is not detected on CT. A high index of suspicion and further exploration are required in order not to miss other vital findings.

## 1. Introduction

Gallstone ileus (GSI) is a rare form of bowel obstruction that occurs in 0.4–1.5% of patients with cholelithiasis [1]. Gallstones (>2 cm) are thought to enter the duodenum through a cholecystoduodenal fistula which forms as a rare complication of gallstone disease. GSI is an important complication of gallstone disease since it is often underreported and poorly managed.

Gallstones which enter the GI tract through a cholecystoduodenal fistula often become mechanically obstructed at the ileum due to its reduced diameter and the ileocecal valve. Patients present with small bowel obstruction symptoms including severe abdominal pain, nausea, vomiting, and abdominal distention. Differential diagnosis is completed by CT scans that reveal the presence of calcified gallstones in the lumen of the bowel at the transition point. Treatment includes minimally invasive surgery if possible or laparotomy to remove the stones and resect bowel if indicated. We report an interesting case of recurrent GSI that was not detected by CT scan but diagnosed intraoperatively. The clinical presentation, investigative findings, and management are discussed along with relevant literatures.

## 2. Case Report

**Figure 1:**
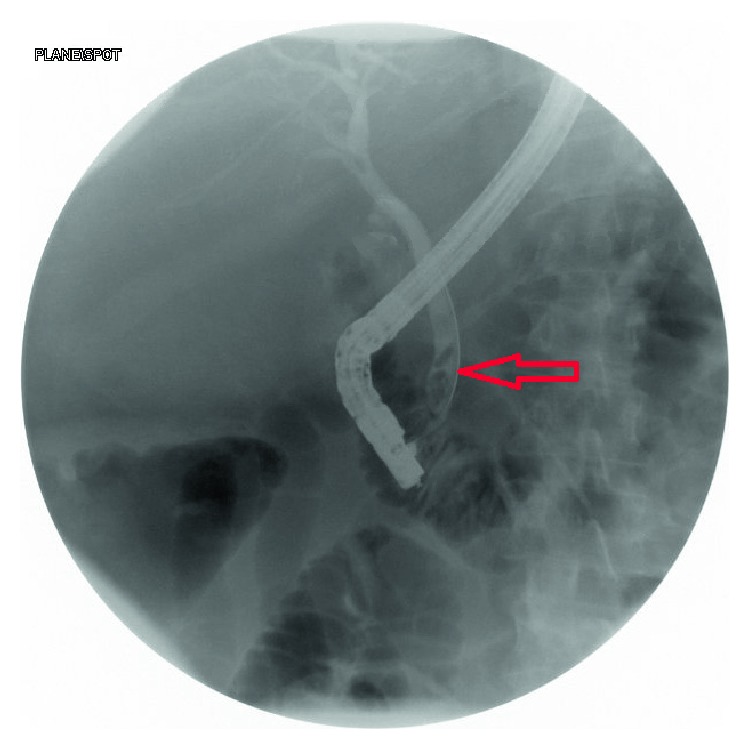
ERCP shows multiple stones in the common bile duct (red arrow).

**Figure 2:**
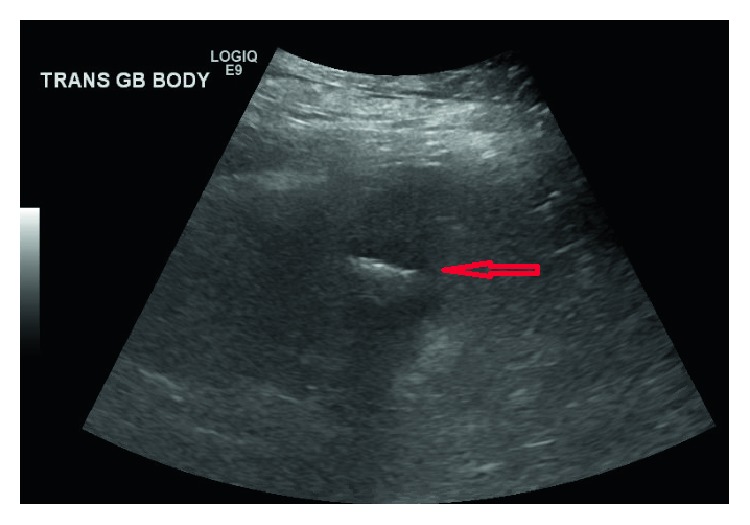
Abdominal ultrasound depicting a large stone in the gallbladder (red arrow).

**Figure 3:**
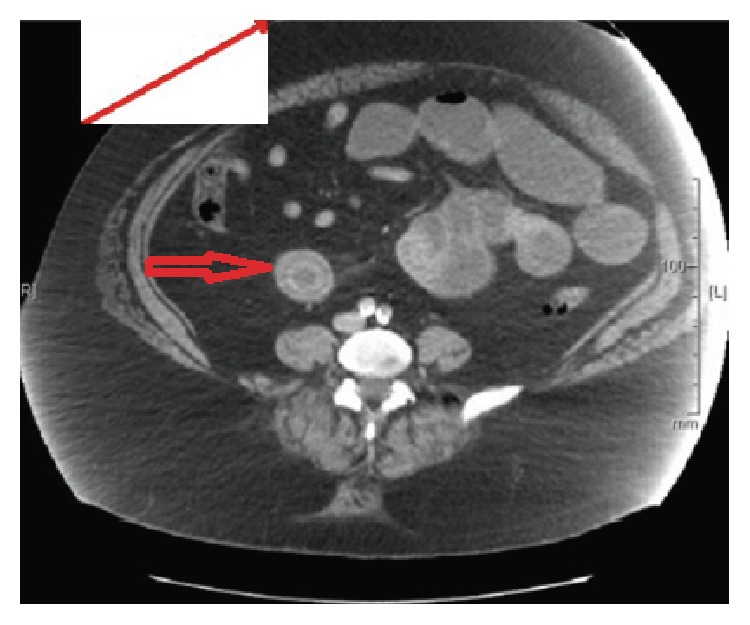
Contrast CT scan depicting gallstone in bowel (short red arrow).

**Figure 4:**
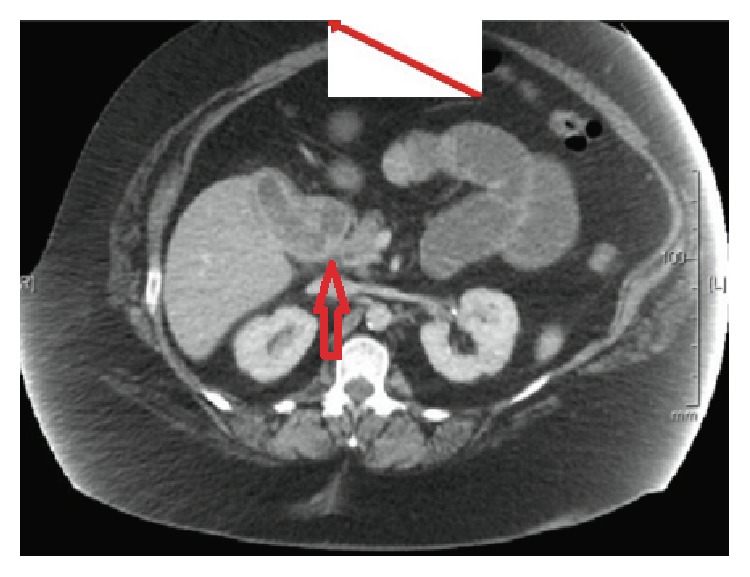
Contrast CT scan depicting cholecystoduodenal fistula (short red arrow).

**Figure 5:**
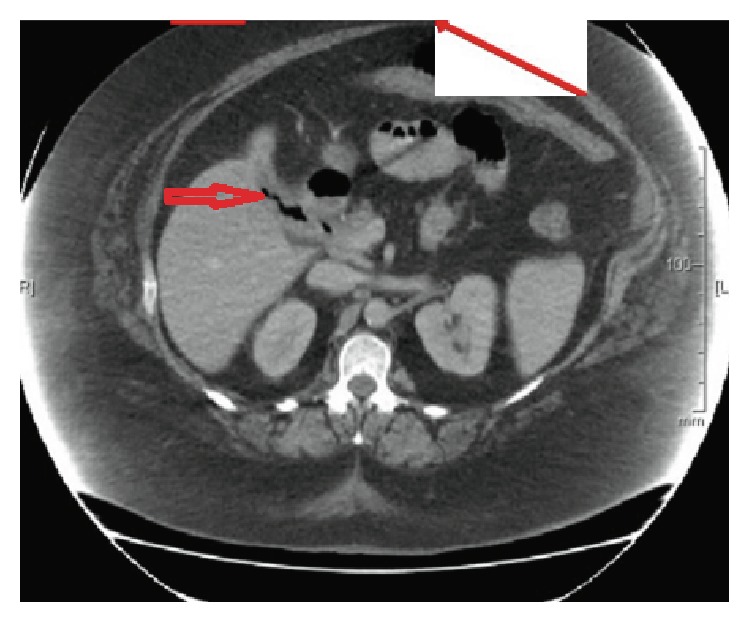
CT contrast scan depicting decompressed GB and air in GB, verifying that a stone was released (short red arrow).

**Figure 6:**
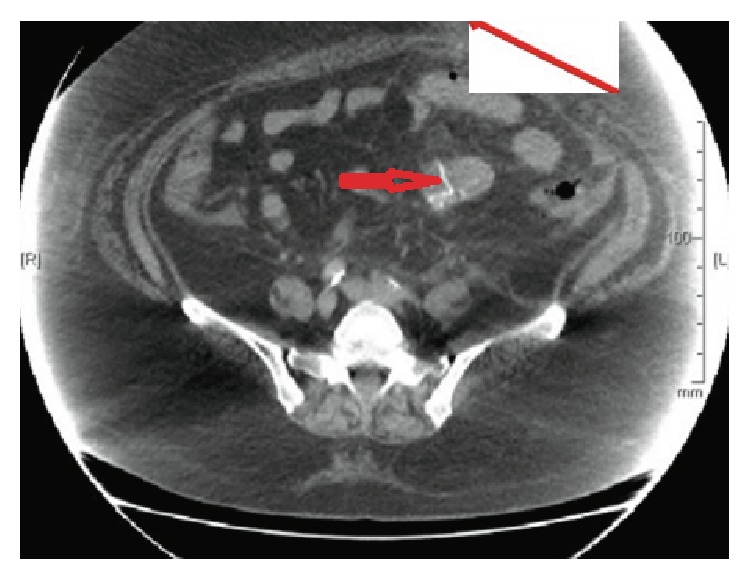
CT contrast scan depicting bowel anastomosis after 1st surgery (short red arrow).

A forty nine-year-old female presented to the emergency department due to episodes of sudden cramping, epigastric pain and nausea 2 months following endoscopic retrograde cholangiopancreatography (ERCP) where three gallstones were removed from the common bile ducts (Figure 1). Before she underwent ERCP and endoscopic sphincterotomy, right upper quadrant abdominal ultrasound done on account of obstructive jaundice showed a noninflammed gallbladder with a large stone and a dilated common bile duct (Figure 2). Her past medical history was also notable for severe aortic stenosis, pulmonary hypertension, diabetes, unstable angina, and morbid obesity with BMI of 60 kg/m^2^. Given her comorbidities, the plan during her ERCP visit was to perform her cholecystectomy at the same time she will undergo her gastric bypass, after optimizing her cardiopulmonary disease. At presentation, her abdominal computed tomography (CT) revealed evidence of small bowel obstruction with clear evidence of gallstone ileus (Figure 3). A cholecystoduodenal fistula was also present (Figure 4). Evidence of a decompressed gallbladder (Figure 5) and a clear transition point were noted. She was taken to the OR for laparoscopic exploration and small bowel examination. The segment that had the stone was identified and exteriorized through a small midline incision; the bowel was necrotic due to pressure ischemia; thus segmental bowel resection was performed with stapled anastomosis (Figure 6). A large, calcified 3.5 cm × 3 cm gallstone was found in the small bowel impacted at 60 cm proximal to the ileocecal valve (Figure 3) with evidence of pressure necrosis to the bowel. The bowel was examined via laparoscopy from the Ligament of Treitz to the ileocecal valve with no evidence of additional stones. Patient recovered appropriately after surgery and was discharged home.

A month later, she presented with signs and symptoms of small bowel obstruction. On physical exam, her abdomen was distended but with no tenderness. Patient was also noted to be in septic shock requiring vasopressors and unable to tolerate surgery; she was treated conservatively with Gastrografin challenge test (50 ml of water mixed with 100 ml of Gastrografin and given through the nasogastric tube, followed by abdominal X-ray in eight hours), which revealed contrast in the colon. Her symptoms completely resolved; she tolerated oral intake and was discharged from the hospital.

**Figure 7:**
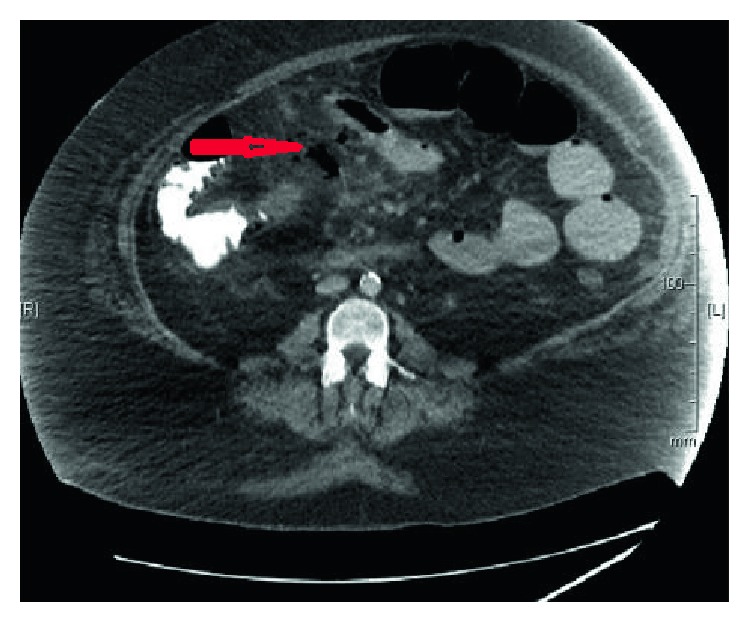
CT contrast scan depicting free air in pocket of abscess where bowel was perforated (short red arrow).

Patient presented again one month later with similar symptoms. At this presentation, she had an abdominal CT scan, which showed dilated bowel with no clear transition point. She was treated conservatively for five days with mild improvement where she tolerated liquids but not solid food. During the same hospital stay, she developed sudden severe abdominal pain, shortness of breath, tachycardia, and hypoxia. Another CT scan was performed with pulmonary embolism (PE) protocol which was negative for PE, but positive for free air (Figure 7). Patient was taken back to the OR for laparotomy, which revealed a second large non-calcified gallstone in the ileum impacted at the ileocecal valve distal to the previous anastomosis and perforated necrotic small bowel with a pocket of abscess. An intraoperative diagnosis of recurrent GSI was made. The abdomen was washed out; bowel resected; but anastomosis could not be performed given the severe inflammation. Patient was brought back to the OR for washout and closure; again, bowel anastomosis could not be performed, nor could the bowel be brought out as an end ileostomy due to severe obesity and shortened thickened mesentery. The bowel was placed in the upper part of the midline incision and the wound closed around it. Patient recovered appropriately and was discharged home. Subsequently, patient underwent elective surgery for cholecystectomy, takedown and primary repair of cholecystoduodenal fistula, bile duct exploration, suture repair of extra hepatic bile duct, and takedown of ileostomy with primary anastomosis.

## 3. Discussion

Gallstone ileus (GSI) is defined as bowel obstruction resulting from the impaction of one or more gallstones due to a cholecystoduodenal fistula. It is an uncommon complication of cholecystolithiasis; a rare cause of obstruction; and accounts for 1% to 4% of cases of mechanical obstruction of the bowel [2]. There are six factors known to cause a fistula: foreign body, radiation, inflammation, epithelialization, neoplasia, and distal obstruction. Risk factors for cholecystolithiasis include obesity and middle age, and it is also noted that females carry a greater risk.

Enhanced abdominal CT with contrast is considered the most helpful tool for diagnosis of gallstone ileus, as it is highly sensitive, specific, and accurate [3]. The findings of CT include obstruction of the small intestine, ectopic gallstones, pneumobilia, and gallbladder abnormalities. The information obtained with CT such as the site of obstruction, the size of the impacted gallstone, and the migration of the gallstone are important for determining whether conservative treatment is appropriate. The goals of treating GSI should be to relieve the intestinal obstruction quickly and to minimize morbidity and mortality. Once a gallstone has impacted within the small bowel and caused ileus, it rarely passes out spontaneously through the intestine [4, 5], though reports indicate that gallstones smaller than 25 mm usually pass through spontaneously [6].

The surgical dilemma in this case lies in the fact that despite the occurrence of a second bowel obstruction, GSI was not clearly noted in the CT scan. In addition, a decompressed gallbladder was not noted as well. It is accepted that the second stone was not calcified which is why it was not detected on the CT scan. It is recommended that these be checked upon initial surgery to prevent recurrent gallstones. Although the bowel was examined in the initial surgery for presence of additional stones in this case, it is probable that the second stone was in the gallbladder or duodenum. While the limitations of bowel examination by laparoscopy cannot be discounted, laparotomy was avoided in the initial surgery due to her significant cardiopulmonary disease and BMI. Recurrence of GSI can happen if the cholecystoduodenal fistula has not been closed, and this is more likely if a second stone is present in the gallbladder. It is fairly controversial in the literature whether to proceed with cholecystectomy or not in the presence of a cholecystoduodenal fistula, but there are substantial data to avoid cholecystectomy due to the high morbidity, though it is not considered a contraindication [7, 8]. Recurrence of GSI has been reported in 5–8% of patients [9], and 50% of all recurrences have been reported to occur within one month of the initial operation, while the remainder do so within 2 years [1, 10]. Methods to prevent recurrence involve cholecystectomy to prevent further bowel impaction, and closure of cholecystoduodenal fistula. In this case, the presence of gallstones after initial endoscopic management of CBD stones should have prompted laparoscopic cholecystectomy before considering a bariatric operation, but this could not be done at the time due to the patient's severe comorbidities. Additionally, the symptoms of small bowel obstruction that occurred one month after GSI laparoscopic management should have led to consideration of a priority treatment of the cholecystoduodenal fistula, but the cholecystoduodenal fistula was not addressed during that presentation because the patient was in septic shock on vasopressors and unable to tolerate surgery. In three cases previously described in the literature, the cholecystoduodenal fistula was completely mobilized with a combination of blunt and sharp dissection and divided using the endoscopic linear stapling device [11]. In two other cases, after division of the cystic duct and cystic artery, the gallbladder was dissected from the liver bed, leaving just the fistulous connection to the duodenum [11]. Then division of the fistula was completed using the same stapling device. All five patients had uneventful postoperative course. The hospital stay of the five patients ranged from 5 to 10 days [11].

## 4. Conclusion

Enhanced abdominal CT remains the most helpful diagnostic tool for GSI. Disproportionate symptoms and signs of a small bowel obstruction, in a patient with a history of gallstone disease or previous GSI, should alert the surgeon to suspect GSI as a possible cause despite the absence of radiologic findings. Further exploration is indicated to detect the presence of other vital findings such as noncalcified gallstones in the small bowel, pressure necrosis, bowel perforation, and abscess formation. Surgery is often indicated to relieve intestinal obstruction quickly and to minimize morbidity and mortality.

## Abbreviations

GSI: Gallstone ileus
SBO: Small bowel obstruction
CT: Computed tomography
ERCP: Endoscopic retrograde cholangiopancreatography
BMI: Body mass index
PE: Pulmonary embolism.

## References

[1] Apollos J. R., Guest R. V. (2015). Recurrent gallstone ileus due to a residual gallstone. A case report and literature review. *International Journal of Surgery Case Reports*.

[2] Ayantunde A. A., Agrawal A. (2007). Gallstone ileus: diagnosis and management. *World Journal of Surgery*.

[3] Yu C. Y., Lin C. C., Shyu R. Y. (2005). Value of CT in the diagnosis and management of gallstone ileus. *World Journal of Gastroenterology*.

[4] Farooq A., Memon B., Memon M. A. (2007). Resolution of gallstone ileus with spontaneous evacuation of gallstone. *Emergency Radiology*.

[5] Miyasaka T., Yoshida H., Makino H., Watanabe M., Uchida E., Uchida E. (2014). Response of gallstone ileus to conservative therapy. *Journal of Nippon Medical School*.

[6] Kasahara Y., Umemura H., Shiraha S., Kuyama T., Sakata K., Kubota H. (1980). Gallstone ileus. Review of 112 patients in the Japanese literature. *The American Journal of Surgery*.

[7] Carlei F., Lezoche E., Lomanto D. (1997). Cholecystoenteric fistula is not a contraindication for laparoscopic cholecystectomy: report of five cases treated by laparoscopic approach. *Surgical Laparoscopy Endoscopy*.

[8] Moreno Ruiz F. J., del Rey Moreno A., Suescun Garcia R. M. (2001). Treatment of cholecystoduodenal fistula in the era of laparoscopy. *Revista Española de Enfermedades Digestivas*.

[9] Hayes N., Saha S. (2012). Recurrent gallstone ileus. *Clinical Medicine & Research*.

[10] Doogue M. P., Choong C. K., Frizelle F. A. (1998). Recurrent gallstone ileus: underestimated. *Australian and New Zealand Journal of Surgery*.

[11] Latic A., Latic F., Delibegovic M., Samardzic J., Kraljik D., Delibegovic S. (2010). Succcessful laparoscopic treatment of cholecystoduodenal fistula. *Medicinski Arhiv*.

